# Antibiotics Used in Empiric Treatment of Ocular Infections Trigger the Bacterial Rcs Stress Response System Independent of Antibiotic Susceptibility

**DOI:** 10.3390/antibiotics10091033

**Published:** 2021-08-25

**Authors:** Nathaniel S. Harshaw, Nicholas A. Stella, Kara M. Lehner, Eric G. Romanowski, Regis P. Kowalski, Robert M. Q. Shanks

**Affiliations:** Charles T. Campbell Ophthalmic Microbiology Laboratory, Department of Ophthalmology, University of Pittsburgh School of Medicine, Pittsburgh, PA 15213, USA; nah126@pitt.edu (N.S.H.); nas92@pitt.edu (N.A.S.); kmlehner@udel.edu (K.M.L.); romanowskieg@upmc.edu (E.G.R.); kowalskirp@upmc.edu (R.P.K.)

**Keywords:** Enterobacterales, keratitis, infection, cornea, bacteria, stress response system, antibiotic

## Abstract

The Rcs phosphorelay is a bacterial stress response system that responds to envelope stresses and in turn controls several virulence-associated pathways, including capsule, flagella, and toxin biosynthesis, of numerous bacterial species. The Rcs system also affects antibiotic tolerance, biofilm formation, and horizontal gene transfer. The Rcs system of the ocular bacterial pathogen *Serratia marcescens* was recently demonstrated to influence ocular pathogenesis in a rabbit model of keratitis, with Rcs-defective mutants causing greater pathology and Rcs-activated strains demonstrating reduced inflammation. The Rcs system is activated by a variety of insults, including β-lactam antibiotics and polymyxin B. In this study, we developed three luminescence-based transcriptional reporters for Rcs system activity and used them to test whether antibiotics used for empiric treatment of ocular infections influence Rcs system activity in a keratitis isolate of *S. marcescens*. These included antibiotics to which the bacteria were susceptible and resistant. Results indicate that cefazolin, ceftazidime, polymyxin B, and vancomycin activate the Rcs system to varying degrees in an RcsB-dependent manner, whereas ciprofloxacin and tobramycin activated the promoter fusions, but in an Rcs-independent manner. Although minimum inhibitory concentration (MIC) analysis demonstrated resistance of the test bacteria to polymyxin B and vancomycin, the Rcs system was activated by sub-inhibitory concentrations of these antibiotics. Together, these data indicate that a bacterial stress system that influences numerous pathogenic phenotypes and drug-tolerance is influenced by different classes of antibiotics despite the susceptibility status of the bacterium.

## 1. Introduction

*Serratia marcescens* is a Gram-negative pathogen from the order Enterobacterales that causes contact lens-associated keratitis in healthy patients [1,2,3] and a wide variety of nosocomial infections in the immune compromised, such as ventilator-associated pneumonia and sepsis in adults and neonates [4,5]. *S. marcescens* isolates are typically resistant to antibiotics of the macrolide, tetracycline, β-lactam, and narrow spectrum cephalosporin classes due to expression of efflux pumps and β-lactamases [6]. However, they are generally susceptible to aminoglycoside, third generation cephalosporin, and fluoroquinolone antibiotics [6,7].

**Figure 1:**
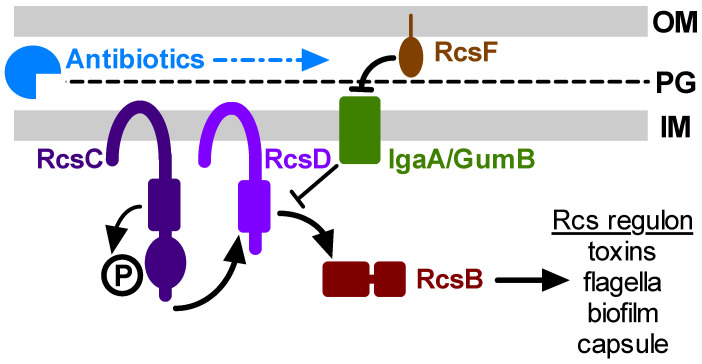
Model for antibiotic activation of the Rcs system. This simplified depiction of the core Rcs system shows the major components required for Rcs function. The Rcs system is a complex phosphorelay signal transduction system that regulates the transcription of many genes through control of the RcsB transcription factor. The IgaA/GumB inner membrane protein blocks Rcs activity under non-stressful conditions. Envelope stress by antibiotics, transmitted by RcsF, prevents IgaA/GumB inhibition of RcsC-D and allows RcsB-mediated transcription. Mutation of *igaA*/*gumB* constitutively derepresses the Rcs transcriptional cascade, and mutation of *rcsB* prevents Rcs system function. This model predicts that Rcs activation by antibiotics can stimulate pathogenesis and antibiotic tolerance phenotypes. OM: outer membrane; PG: peptidoglycan; IM: inner membrane.

The Rcs stress response system has been found in bacteria from the Enterobacterales including, but not limited to, numerous pathogens, such as *Escherichia coli*, *Klebsiella* species, *Proteus mirabilis*, *Salmonella enterica*, and *Yersinia pestis* [8]. The core Rcs system (Figure 1) is a complex signal transduction cascade composed of a variety of components that include outer membrane protein RcsF, inner membrane protein IgaA, two histidine kinase-related proteins, RcsC and RcsD, and the response regulator transcription factor RcsB [8]. Rcs signaling occurs in response to cell envelope stresses, such as defects in peptidoglycan and lipopolysaccharide (LPS) structure, perturbations of the outer membrane β-barrel protein assembly complex, and lipoprotein trafficking [8,9]. Antimicrobials known to activate the Rcs system, mostly from studies with *E. coli* and *S. enterica*, include polymyxin B [10] and other antimicrobial peptides [11], and cell wall-targeting β-lactam and cephalosporin antibiotics [9,12]. However, this has not been tested in ocular pathogens such as *S. marcescens*.

Importantly, the Rcs system has been shown to contribute to antibiotic tolerance by a number of bacteria, with Rcs system-defective mutants being more susceptible to penicillin and cephalosporin antibiotics for *E. coli* [12] and polymyxin B for *E. coli* [13] and *S. enterica* [10]. Similarly, induced expression of *rcsB* or expression of alleles that increase Rcs activity conferred increased tolerance to β-lactam and cephalosporin antibiotics for *E. coli* [12,14]. A major mechanism used by bacteria to increase antibiotic tolerance is biofilm formation [15]. The Rcs system plays a positive role in *S. marcescens* biofilm formation under high sheer conditions by promoting capsular polysaccharide synthesis [16]. A similar role for the Rcs system in *E. coli* and *S. enterica* biofilm formation has been described [17]. Beyond antibiotic tolerance, a recent study by Smith et al. suggests that Rcs plays a role in the acquisition of genetic elements by *Serratia* sp. 39006 that may contribute to horizontal gene transfer and antibiotic resistance [18].

The *S. marcescens* Rcs system has been shown to regulate synthesis of the ShlA cytolysin [19,20], where it is also a key regulator of capsular polysaccharide and flagella synthesis, as well as the production of a hemolytic biosurfactant [16,21]. Importantly, the Rcs system was shown to be a major regulator of *S. marcescens* ocular pathogenesis [22].

The goal of this study was to evaluate whether antibiotics commonly used topically for empiric treatment of ocular infections activate the bacterial Rcs pathway. In this study, we used antibiotics recommended for the empiric treatment of bacterial keratitis by the American Academy of Ophthalmology [23]. Given the role of the Rcs system in promoting antibiotic tolerance and the regulation of virulence factors, it is possible that activation of this system could influence clinical outcomes for patients infected by the Enterobacterales. To that end, we developed luminescent reporter plasmids for Rcs activity and used them in a keratitis isolate of *S. marcescens* with antibiotics from several classes that are recommended for the treatment of ocular infections.

## 2. Results

### 2.1. Generation of Luminescent Reporter Plasmids for Rcs System Activity

**Figure 2:**
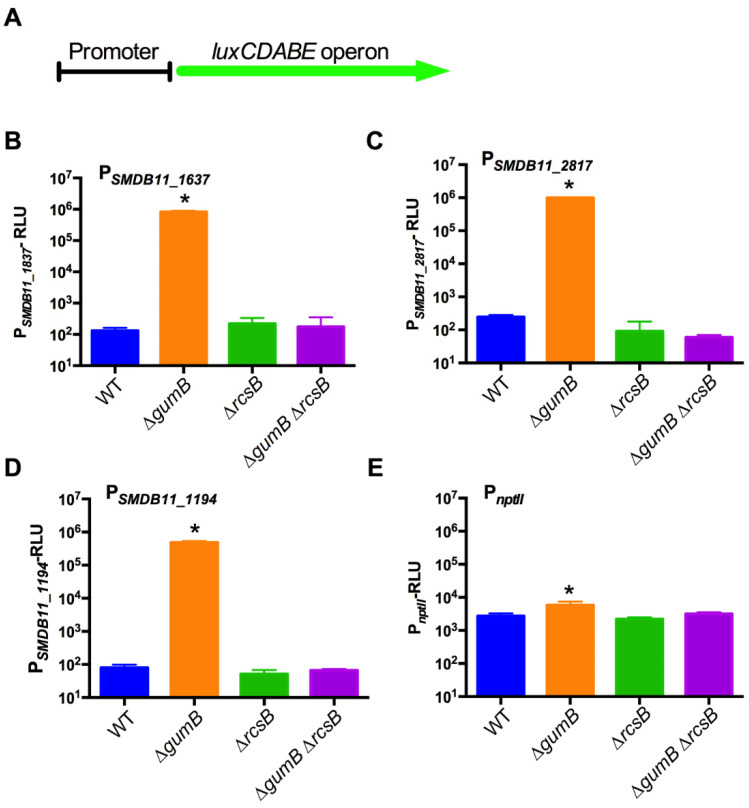
Validation of Rcs-responsive transcriptional reporter plasmids. (**A**). Schematic diagram of a promoter transcriptional fusion to the luminescence-producing *luxCDABE* operon that was cloned into a broad-host range medium-copy plasmid. Four different promoters were evaluated by moving them into *S. marcescens* with normal (WT), hyper-activated (∆*gumB*), or defective (∆*rcsB*, ∆*gumB* ∆*rcsB*) Rcs-systems. (**B**–**E**). Transcription from the four promoters was measured using a luminometer after the bacteria were grown for 20 h in LB medium (*n* = 4–6). The luminescence values were normalized by optical density, which was similar for each genotype. The *nptII* promoter is an *E. coli* promoter that was used as a constitutive control. The PSMDB11_1637, PSMDB11_2817, and PSMDB11_1194 promoters were Rcs responsive. The asterisks (*) indicate that the ∆*gumB* group is statistically different than the other groups, *p* < 0.01. WT: wild type.

In order to conveniently measure Rcs activation, luminescent reporter plasmids were made using Rcs-responsive promoters. GumB, an IgaA ortholog, is a negative regulator of Rcs activity, such that a *gumB* deletion mutant has a highly activated Rcs system [16,19]. Transcriptomic analysis of a ∆*gumB* mutant was used to identify genes that were more highly expressed than in the wild type (to be described elsewhere). Three promoters were cloned upstream of the *luxCDABE* operon on a broad-host range low-copy vector (Figure 2A and Figure S1). The promoters were for the SMDB11_1637, SMDB11_2817, and SMDB11_1194 open reading frames. All of these previously uncharacterized open reading frames bear high similarity to Rcs-regulated genes in other bacteria. SMDB11_1637 is similar to osmotically inducible lipoprotein B (*osmB*), which is positively regulated by the Rcs system in *Erwinia amylovora* [24], *E. coli* [25,26], *P. mirabilis* [27], *S. enterica* [28], and *Yersinia pseudotuberculosis* [29]. SMDB11_1194 is highly similar to *umoD*, which is Rcs-regulated in *P. mirabilis* [27], as is its ortholog YPO1624 in *Y. pseudotuberculosis*. SMDB11_2817 has similarity to *yaaX* from *E. coli* with the DUF2502 domain of unknown function and was identified as an RcsB-regulated gene in *E. coli* [25]. 

In addition, the *nptII* promoter from the Tn5 transposon was used as a constitutive control promoter to test for theoretical physiological conditions that could interfere with luminescence. 

To validate the Rcs system activation of these promoters, they were moved into a contact lens-associated keratitis wild-type (WT) isolate of *S. marcescens*, strain K904, and isogenic mutants with manipulated Rcs systems that confer high (∆*gumB*) or no (∆*rcsB* and ∆*gumB* ∆*rcsB*) Rcs activity. Strains and plasmids are listed in Table S1 (Supplementary Materials). Bacteria were grown overnight, and the luminescence was determined as a function of culture optical density (Figure 2). The test strains were previously shown to achieve similar growth levels over the tested time frame [30].

The results (Figure 2) suggested that the promoter activity for each of the genes, except the control *nptII* promoter, was highly increased (>6000-fold) in the Rcs-activated mutant background (∆*gumB*). Furthermore, a clear reduction in luminescence was observed in the ∆*gumB* ∆*rcsB* double mutant, confirming that the increase observed in the ∆*gumB* mutant was Rcs dependent. There was some expression in the absence of Rcs activity (see ∆*rcsB* mutant), indicating that there is some Rcs-independent expression from these promoters (i.e., other transcription factors may regulate some of these promoters; see discussion). Importantly, the *nptII* promoter showed only a minor but significant change (~2-fold) among the various mutant backgrounds, suggesting that the Rcs system status has little to no impact on the ability of the luminescent reporter system to function. Together, these results indicate that we have identified and cloned three Rcs system-responsive promoters and created reporter constructs to analyze compounds that may influence Rcs-activity.

### 2.2. Antibiotics Targeting the Cell Envelope Activate the S. marcescens Rcs System Regardless of the Antibiotic Susceptibility Status of the Bacterium

The antibiotic susceptibility of four antibiotics used in treatment of ocular infections was analyzed: polymyxin B, cefazolin, ceftazidime, and vancomycin (Table 1 and Table 2). These target either the peptidoglycan cell wall or the bacterial membrane. Minimum inhibitory concentrations (MICs) for the antibiotics to inhibit *S. marcescens* strain K904 were determined (Table 2). The isolate was susceptible to ceftazidime, but was able to grow at the highest tested concentrations of polymyxin B, cefazolin, and vancomycin. This was a typical pattern for keratitis isolates of *S. marcescens* [7]. Nevertheless, prior to identification of the infecting microbe, any of the antibiotics other than polymyxin B are candidates for empiric therapy for keratitis. 

Polymyxin B was previously shown to activate the Rcs system of *E. coli* and *S. enterica* [11,13]. Unlike these bacteria, the vast majority of *S. marcescens* isolates are resistant to polymyxin B due to a 4-aminoarabinose modification of the lipid A portion of the lipopolysaccharide molecules that populate the outer leaflet of the outer membrane [38]. The K904 strain was evaluated for polymyxin B susceptibility and found to be resistant (MIC > 1024, Table 2). The induction of the Rcs system by polymyxin B in a resistant bacterial species has not been evaluated. 

**Figure 3:**
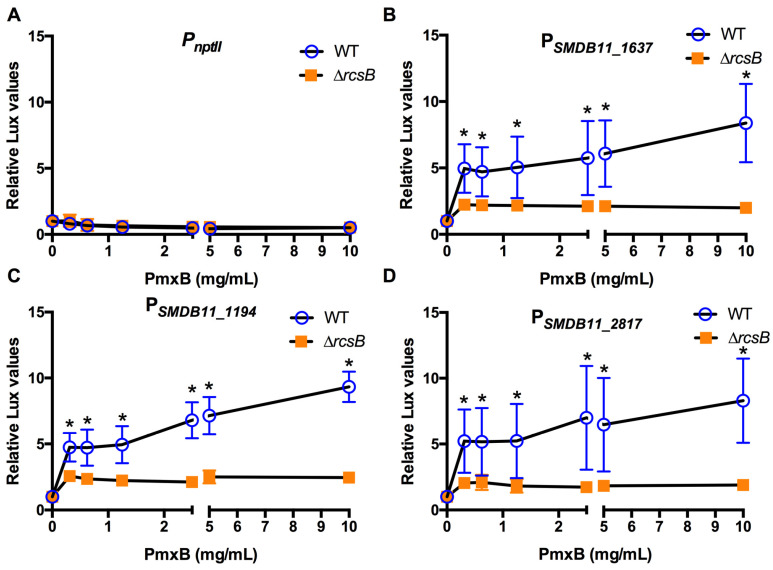
Effect of cell envelope-targeting antibiotic polymyxin B on Rcs-activated promoters (**A**–**D**). Relative luminescence values were determined by dividing luminescence by optical density after 4 h of antibiotic challenge. The *nptII* promoter (**A**) was unaffected by polymyxin B; however, the Rcs-dependent promoters (**B**–**D**) were activated to a greater extent in the WT than the Rcs-defective ∆*rcsB* mutant. Mean and standard deviation are shown (*n* = 6–9 are shown). Asterisks (*) indicate statistical differences between groups at the indicated concentrations, *p* < 0.05.

Polymyxin B did not activate the *nptII* promoter in the WT bacteria, as expected (Figure 3A); however, the Rcs-dependent promoters were activated in an antibiotic dose-dependent manner, up to 5–10 fold above the absence of antibiotic (Figure 3B–D). To ensure that the effect was Rcs-dependent, the reporters were tested in an isogenic ∆*rcsB* mutant strain. While there was a less than 2-fold increase in luminescence correlating with the presence of antibiotics, it was not dose dependent in the ∆*rcsB* mutant (Figure 3B–D). These suggest that polymyxin B activates the Rcs system even in a resistant bacterium.

**Figure 4:**
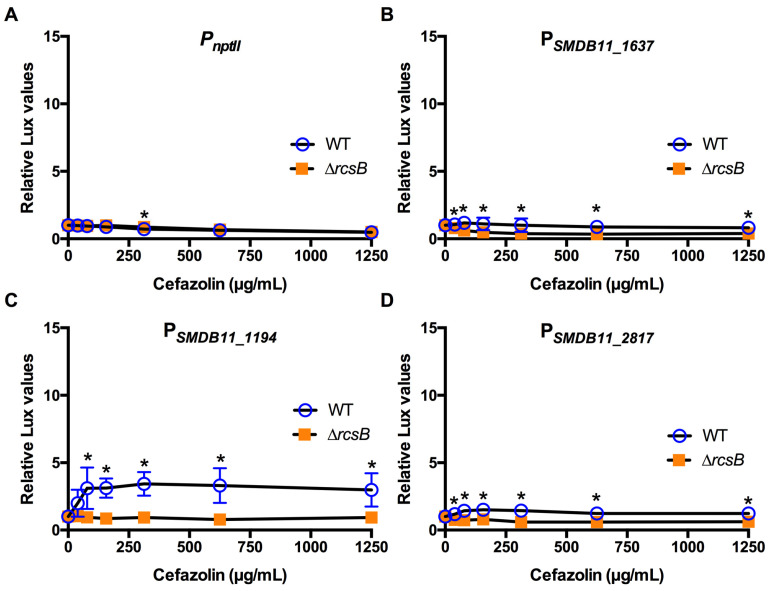
Effect of cell wall activating cefazolin on Rcs-activated promoters (**A**–**D**). Relative luminescence values were determined by dividing luminescence by optical density after 4 h of antibiotic challenge. The *nptII* promoter (**A**) was unaffected by cefazolin. Only the Rcs-dependent SMDB11_1194 promoter (**C**) was activated to a greater extent in the WT than the Rcs-defective ∆*rcsB* mutant. Mean and standard deviation are shown (*n* = 6–9 are shown). Asterisks (*) indicate statistical differences between groups at the indicated concentrations, *p* < 0.05.

**Figure 5:**
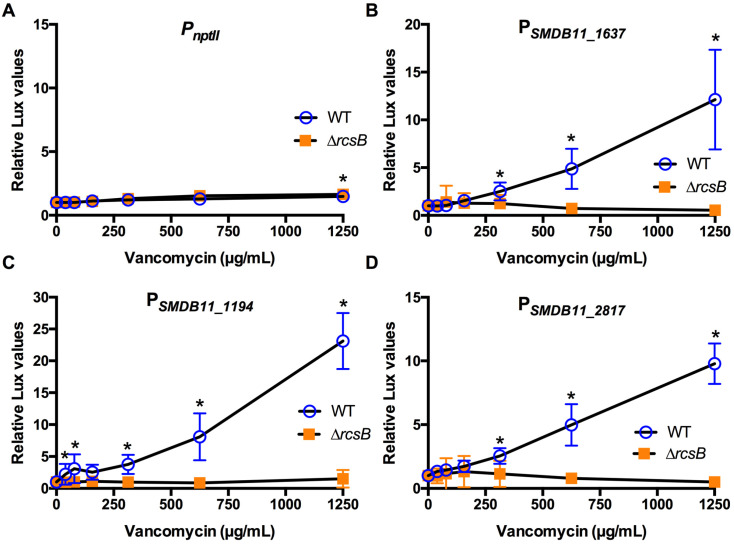
Effect of the cell wall activating antibiotic vancomycin on Rcs-activated promoters (**A**–**D**). Relative luminescence values were determined by dividing luminescence by optical density after 4 h of antibiotic challenge. The *nptII* promoter (**A**) was unaffected by vancomycin. The experimental promoters (**B**–**D**) were activated to a greater extent in the WT than the Rcs-defective ∆*rcsB* mutant. Mean and standard deviation are shown (*n* = 6–9 are shown). Asterisks (*) indicate statistical differences between groups at the indicated concentrations, *p* < 0.05.

**Figure 6:**
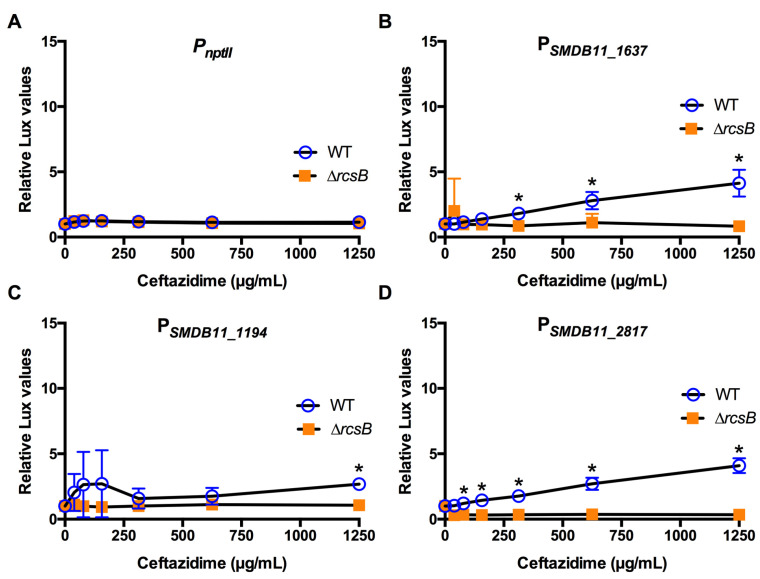
Effect of the cell wall activating antibiotic ceftazidime on Rcs-activated promoters (**A**–**D**). Relative luminescence values were determined by dividing luminescence by optical density after 4 h of antibiotic challenge. The *nptII* promoter (**A**) was unaffected by ceftazidime. The Rcs-dependent promoters (**B**–**D**) were activated to a greater extent in the WT than the Rcs-defective ∆*rcsB* mutant. Mean and standard deviation are shown (*n* = 6–9 are shown). Asterisks (*) indicate statistical differences between groups at the indicated concentrations, *p* < 0.05.

The identical approach was used for three different classes of cell wall-targeting antibiotics. A β-lactam antibiotic, cefazolin, is used to treat Gram-positive bacteria (Table 1). *S. marcescens* strain K904 was resistant to cefazolin (Table 2). Experiments indicated very little induction except in the SMDB11_1194 promoter (Figure 4). Similarly, *S. marcescens* K904 was resistant to the glycopeptide vancomycin (Table 1 and Table 2) and was activated by the three Rcs-dependent promoters in the WT but not the ∆*rcsB* mutant (Figure 5). By contrast, *S. marcescens* was susceptible to the cephalosporin ceftazidime (Table 1 and Table 2). Two of the Rcs-dependent promoters were activated by ceftazidime in the WT but not the ∆*rcsB* mutants (Figure 6).

### 2.3. Non-Cell Envelope-Targeting Antibiotics Activated the Test Promoters in an Rcs-Independent Manner

**Figure 7:**
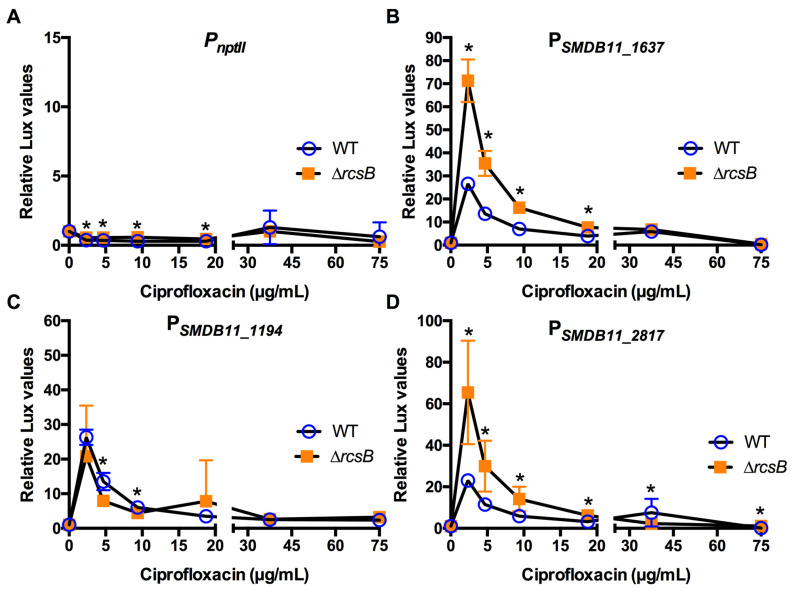
Effect of DNA metabolism-targeting ciprofloxacin on Rcs-activated promoters (**A**–**D**). Relative luminescence values were determined by dividing luminescence by optical density after 4 h of antibiotic challenge. The *nptII* promoter (**A**) was largely unaffected by ciprofloxacin. The experimental promoters (**B**–**D**) were activated to an equal or greater extent in the ∆*rcsB* mutant than the WT. Mean and standard deviation are shown (*n* = 6–9 are shown). Asterisks (*) indicate statistical differences between groups at the indicated concentrations, *p* < 0.05.

The same approach used for envelope-targeting antibiotics was used for two non-envelope-targeting antibiotics. Ciprofloxacin is a fluoroquinolone that targets DNA metabolism and is highly effective against Gram-negative ocular pathogens such as *Pseudomonas aeruginosa* and *S. marcescens* (Table 1 and Table 2). Figure 7 demonstrates that the three test promoters were highly activated by low concentrations of ciprofloxacin in the WT. However, similar, or even higher levels of expression, were observed in the Rcs-defective mutant, indicating that the activation of the test promoters was Rcs-independent and suggesting that ciprofloxacin does not activate the Rcs system.

**Figure 8:**
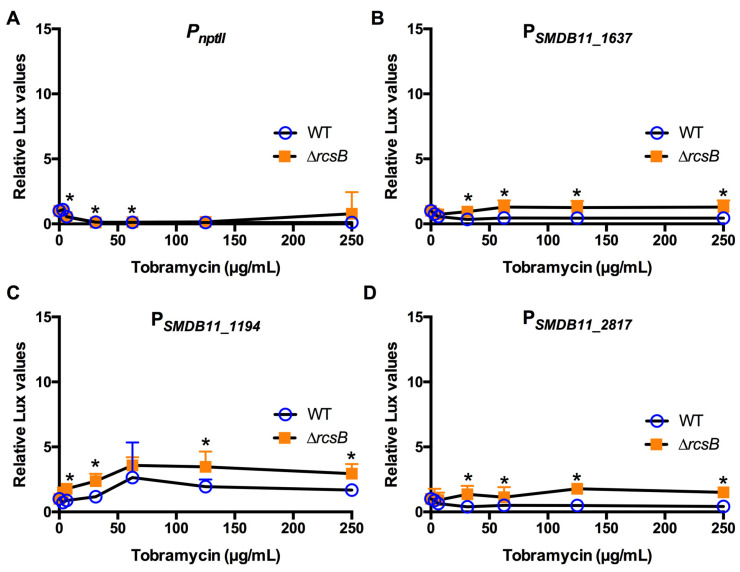
Effect of protein synthesis-targeting antibiotic tobramycin on Rcs-activated promoters (**A**–**D**). Relative luminescence values were determined by dividing luminescence by optical density after 4 h of antibiotic challenge. The *nptII* promoter (**A**) was unaffected by tobramycin. The experimental promoters were expressed to equal or greater extent in the ∆*rcsB* mutant than the WT. Mean and standard deviation are shown (*n* = 6–9 are shown). Asterisks (*) indicate statistical differences between groups at the indicated concentrations, *p* < 0.05.

The ribosome-targeting aminoglycoside antibiotic tobramycin is used to treat ocular bacterial pathogens (Table 1 and Table 2). Data in Figure 8 indicate very little induction of promoter activity by tobramycin except by low induction of the SMDB11_1194 promoter. Slightly higher expression of the promoters was observed in the ∆*rcsB* mutant, suggesting that the promoter transcriptional activation was Rcs-independent.

## 3. Discussion

The major impetus behind this study was to test whether the Rcs system was activated by antibiotics used in topical treatment of keratitis. The results show that several of the antibiotics that are widely used for this purpose indeed do activate the Rcs system. A limitation of the study is that the ocular surface antibiotic pharmacokinetics differ from those in the microplate. While topical antibiotics use very high concentrations, the combined action of blinking and the tears wash away most topical antibiotics in a short time frame. Similarly, antibiotic concentrations reduce over time after application, which could lead to levels that activate the Rcs or other stress response systems. Nevertheless, patients with keratitis are given multiple doses of topical antibiotics each day, and although there are limited studies, data demonstrate measurable quantities of the antibiotics accumulate in the corneal tissue [32,36]. Furthermore, experimental studies with rabbits have shown that concentrations of topically applied antibiotics that mimic clinical treatment regimens are able to kill bacteria in the cornea and even to achieve concentrations sufficient to eliminate bacteria that are considered resistant by systemic standards [39,40,41]. Therefore, the combination of the highly sensitive promoters and large antibiotic concentration gradients used in this study likely reflects the antibiotic concentrations that bacteria experience during antibiotic therapy for ocular infections. 

Additional differences between this in vitro study and the ocular environment include a lack of the innate immune system components that could influence the activity of the antibiotics through synergistic effects or produce envelope stress through other means, such as envelope-targeting defensins and enzymes such as lysozyme and phospholipase A [42,43]. These potential effects will be analyzed in future studies.

Data from this study indicate that the promoters for SMDB11 ORF 1194, 1637, and 2817 are Rcs responsive, given the several log_10_-fold increase in the ∆*gumB* mutant that required a functional *rcsB* gene. However, it is clear that the selected promoters could also be strongly activated by ciprofloxacin in an Rcs-independent manner. This is not unexpected, as several envelope stress response systems, beyond Rcs, are conserved among the Enterobacterales. For example, in *Salmonella*, the promoter of the *osmB* gene (similar to SMDB11_1687) is activated by both the Rcs and the RpoS stress response systems [28], suggesting that individual stress response genes are controlled by multiple regulatory systems. The use of the ∆*rcsB* strain in addition to the WT enabled clear identification of Rcs-dependent activation of the reporters by ocular antibiotics. 

Remarkably, even antibiotics that *S. marcescens* strain K904 was highly resistant to, such as polymyxin B and vancomycin, elicited strong activation of the Rcs system. These results suggest that the antibiotics are still capable of perturbing the envelope, even if they are not able to prevent growth. In general, the three different promoters reacted similarly to each antibiotic, with the notable exception of cefazolin, which only activated the SMDB11_1194 promoter. This may be due to differential promoter elements that make this promoter more sensitive than the others to Rcs function. Polymyxin B has been shown to activate the Rcs system in polymyxin B susceptible *S. enterica* at subinhibitory levels, and this was postulated to be driven by polymyxin B’s selective permeabilization of the outer membrane to hydrophobic compounds at low concentrations [11,44]. Several other antibiotics that directly or indirectly affect membrane permeability, including β-lactam, fluoroquinolone, and macrolide antibiotics, are likely capable of the same effect [45].

Of interest, ciprofloxacin appeared to activate some of these promoters to a greater extent in the ∆*rcsB* mutant. This suggests that Rcs may actively inhibit other stress response systems under normal situations. Consistent with this observation, previous studies have demonstrated a complex interplay between the Rcs system and other envelope stress response systems [46,47,48]. Beyond Rcs, there are other envelope sensing stress response systems in the Enterobacterales, including RpoS, the Cpx system, the phage response proteins, EnvZ/OmpR, and others (reviewed by [46,47,48]). Very few studies have evaluated the roles of these proteins in *Serratia* species; however, studies have demonstrated pleiotropic roles for Cpx, OmpR, and RpoS in the control of pathogenesis-relevant phenotypes, such as biofilm formation, and secreted enzymes and cytotoxic secondary metabolite production in *Serratia* species [49,50,51,52]. The activation of the Rcs system, as noted above, is correlated with changes that drive virulence-associated phenotypes, such as biofilm formation [16,17]. The ability of antibiotics to promote these phenotypes through the Rcs system during ocular infections will be evaluated in subsequent studies.

During the course of this study, another group reported on the production of a Rcs-dependent fluorescent reporter system for *E. coli* [9]. This was subsequently and cleverly used to screen small molecule libraries for activators of the Rcs system, with the concept that the identified molecules may be evaluated and developed as envelope-targeting antimicrobials [53]. Therefore, Rcs-reporter systems can be used for both basic biomedical research and applied studies, and the reporters generated in this study could be useful to a variety of researchers. 

## 4. Materials and Methods

### 4.1. Bacterial Growth and Media

Bacteria (Table S1) were maintained in glycerol stocks at −80 °C and streaked out on lysogeny broth (LB) agar [54] before use. Single colonies were grown in LB broth with aeration on a tissue culture rotor (New Brunswick Tc-7, New Brunswick, NJ, USA). Gentamicin (10 µg/mL) was used to maintain plasmids. Plasmids were moved into *S. marcescens* by conjugation, and tetracycline (10 µg/mL) was used for selection against donor *E. coli* [55], as previously described. Antibiotics were obtained from Sigma-Aldrich (St. Louis, MO, USA) unless otherwise noted.

### 4.2. Generation of Luminescence Reporters

The *pigA* promoter on plasmid pMQ713 [56] was replaced with the SMDB11_1194, SMDB11_1637, and SMDB11_2817 using yeast homologous recombination, as previously described [57,58]. Plasmids are listed in Table S1. The pMQ713 plasmid was linearized by restriction enzyme digestion with EcoR1 and Sal1 (New England Biolabs, Ipswich, MA, USA). DNA for the three promoter regions were synthesized as linear double-stranded DNA fragments (Integrated DNA Technologies, Coralville, IA, USA) that include DNA for the promoter region and for site-directed recombination with pMQ713 that places the *luxCDABE* reporter under transcriptional control of the respective promoter (listed in Table S2). The lengths of the cloned promoters were 338 bp for SMDB11_1194, 354 bp for SMDB11_1637, and 337 bp for SMDB11_2817. To generate the *nptII*-driven *luxCDABE* plasmid, the *tdtomato* gene from pMQ414 was digested with BamH1 and EcoR1 enzymes, and the *luxCDABE* operon was amplified by PCR from pMQ670 [59] using primers 3805 and 3806 via PrimeSTAR DNA polymerase (Takara Bio, San Jose, CA, USA). The linearized plasmid and *luxCDABE* amplicon were combined as above. The plasmids were isolated, and the cloned promoter region was sequenced to validate the constructs. 

### 4.3. Luminescent Reporter Assays

Strains of *S. marcescens* bearing luminescent reporter plasmids were taken from a −80 °C freezer and grown on LB agar with tetracycline (10 µg/mL) and gentamicin (10 µg/mL) for 18 h at 30 °C. Single colonies were grown in LB broth with gentamicin in test tubes, which were aerated on a tissue culture rotor for 18–20 h at 30 °C. For reporter verification experiments, the cultures were measured for growth by evaluating optical density at λ = 600 nm (OD_600_) and luminescence at the 527 nm setting from 200 µL samples in black-sided, clear-bottomed 96-well plates (ThermoFisher, Waltham, MA, USA, product 165305) using plate readers (Molecular Devices SpectraMax M3 and L, San Jose, CA, USA). Relative luminescence units (RLU) values were determined by dividing the raw luminescence values by optical density values. 

For antibiotic effect on promoter activity experiments, cultures were normalized by measuring optical density at OD_600_ across a 1-cm path length cuvette with a spectrophotometer (Molecular Devices SpectraMax M3). The assay was conducted in 96-well black-sided, optical bottom plates as above. Two-fold serial dilutions of the antibiotics were performed with a multichannel pipette, and the bacteria were then added to a final concentration of OD_600_ = 0.05 (~9 × 10^7^ CFU/mL). The plate was incubated for 4 h at 37 °C in a plastic bag with a dampened paper towel. At 0 and 4 h, luminescence and OD_600_ values were obtained as above. To obtain RLU values, luminescence values were divided by optical density and normalized to RLU values from the no antibiotic challenge control wells.

The antibiotics and maximum concentrations used in this study were polymyxin B at 10 mg/mL (Sigma, St. Louis, MO, USA, product 5291), vancomycin at 5 mg/mL (Fresenius-Kabi, Bad Homburg, Germany, product C22110), cefazolin at 5 mg/mL (WG Critical Care, Paramus, NJ, USA, product 44567-707-25), ceftazidime at 5 mg/mL (Sigma, product C-3809), tobramycin at 1 mg/mL (XGen Pharmaceuticals, Horseheads, NY, USA, product 39882-0412-1), and ciprofloxacin at 0.3 mg/mL (LKT Labs, St. Paul, MN, USA, product C3262). Stock solutions of antibiotic were prepared in a sterile 15-mL polypropylene centrifuge (Corning, Corning, NY, USA) tube by dissolving the solid antibiotics in lysogeny broth (LB). To ensure sterility, the antibiotic solution was filtered using a PVDF 0.22-μm filter (Millipore SLGVR33RB, Cork, Ireland) into a new sterile polypropylene centrifuge tube. All samples were stored at 4 °C when not in use. The antibiotic gentamicin (10 μg/mL) was added into the assay samples for all trials to maintain the plasmids. 

### 4.4. Minimum Inhibitory Concentration Analysis

MIC values were determined by Epsilometer (E-test) testing (bioMérieux Inc., Durham, NC, USA) for cefazolin, ceftazidime, vancomycin, tobramycin, gentamicin, polymyxin B, and ciprofloxacin. In brief, an overnight growth of bacteria was adjusted to a turbidity standard of 0.5 McFarland (~1.2 × 10^8^ CFU/mL) and overlayed with swab streaking on Mueller Hinton agar. E-test strips were placed onto the agar and allowed to incubate for 24 h at 37 °C. The MIC gradients were visually determined and recorded after incubation following the manufacturer’s guidelines.

### 4.5. Statistical Analysis

Tests were performed using Prism software (GraphPad, San Diego, CA, USA). One-way ANOVA with Tukey’s post-test was used to compare multiple groups and Student’s *t*-test was used to compare between pairs. For this study, *p*-values of less than 0.05 were considered significant.

## 5. Conclusions

In this study, luminescence reporters for Rcs-stress system activation were generated for use in bacteria of the Enterobacterales order. This stress system induces major transcriptional changes in response envelope stresses that result in increased capsule production and biofilm formation. Using these reporters, the Rcs response of the ocular pathogen *S. marcescens* to antibiotics used for the treatment of keratitis was evaluated. Several classes of antibiotics used to treat keratitis induced the Rcs system even when the test bacterium was highly resistant to the respective antibiotic. These data suggest that topical treatment of ocular infections with antibiotics may lead to Rcs-dependent phenotypic changes that aid in bacterial antibiotic tolerance.

## Figures and Tables

**Table 1:** Characteristics of antibiotics used in this study.

Antibiotic	Typical Topical Drug Concentration [23]	Corneal Tissue Concentration	Typical Systemic Dose	Peak Serum Concentration (µg/mL)	Antibiotic Concentration Used in This Study (µg/mL)
Cefazolin	50 mg/mL Fortified [23]	NA	1 g (IV) q8h [31]	200 µg/mL [31]	39–1250
Ceftazidime	50 mg/mL Fortified [23]	NA	2 g (IV) q8h [31]	120 µg/mL [31]	39–1250
Ciprofloxacin	3 mg/mL Commercial [23]	9.92 ± 10.99 μg/g [32]	400 mg (IV) q12h [31] 500–750 mg (PO) q12h [31]	4.6 µg/mL [31] (IV) 2.8 µg/mL [31] (PO)	2.5–75
Polymyxin B	0.75–1 mg/mL (7500–10,000 units/mL) Commercial [33]	NA	1.25 mg/kg (IV) q12h (1 mg = 10,000 units) [31]	8 µg/mL [31]	30–10,000
Tobramycin	3 mg/mL Commercial [34] 9–14 mg/mL Fortified [23]	NA	5 mg/kg (IV) q24h [31] or 240 mg (IV) q24h [31] (preferred over q8h dosing)	16–24 µg/mL q24h dosing [31]	8–250
Vancomycin	10–50 mg/mL [23,35]	46.7 µg/g [36]	1 g (IV) q12h [31]	40 µg/mL [31]	39–1250

NA: information not available; IV: intravenous; PO: per os; q8h: every 8 h; q12h: every 12 h, q24h: every 24 h.

**Table 2:** Antibiotic susceptibility analysis of *S. marcescens* strain K904.

Antibiotic	Class	Target	MIC ^a^—WT (µg/mL)	MIC—∆*rcsB* (µg/mL)	Susceptibility ^b^	Rcs-Specific Induction ^c^
Cefazolin	Cephalosporin	Cell wall	>256, >256	>256, >256	No	Yes
Ceftazidime	Cephalosporin	Cell wall	0.25, 0.19	0.19, 0.19	Yes	Yes
Ciprofloxacin	Fluoroquinolone	DNA gyrase and topoisomerase IV	0.064, 0.064	0.094, 0.470	Yes	No
Polymyxin B	Polymyxin	Cell membrane	>1024, >1024	>1024, >1024	No	Yes
Tobramycin	Aminoglycoside	Ribosome	2, 2	1.5, 1.5	Yes	No
Vancomycin	Glycopeptide	Cell wall	>256, >256	>256, >256	No	Yes

^a^ Minimum inhibitory concentrations (MICs) were determined by E-test; values for two independent tests are shown. ^b^ Susceptibility status was based on Clinical and Laboratory Standards Institute breakpoints [37]. ^c^ At least one promoter was activated in the wild type, but none in the ∆*rcsB* mutant.

## Supplementary Materials

The following are available online at https://www.mdpi.com/article/10.3390/antibiotics10091033/s1: Table S1: *S. marcescens* strains and plasmids used in this study; Table S2: Nucleic acids used in this study; Figure S1: Diagram of pMQ747 used in this study.

## References

[1] Alexandrakis G., Alfonso E.C., Miller D. (2000). Shifting trends in bacterial keratitis in south Florida and emerging resistance to fluoroquinolones. Ophthalmology.

[2] Hume E.B., Willcox M.D. (2004). Emergence of *Serratia marcescens* as an ocular surface pathogen. Arch. Soc. Esp. Oftalmol..

[3] Mah-Sadorra J.H., Najjar D.M., Rapuano C.J., Laibson P.R., Cohen E.J. (2005). *Serratia* corneal ulcers: A retrospective clinical study. Cornea.

[4] Mahlen S.D. (2011). Serratia infections: From military experiments to current practice. Clin. Microbiol. Rev..

[5] Richards M.J., Edwards J.R., Culver D.H., Gaynes R.P. (2000). Nosocomial infections in combined medical-surgical intensive care units in the United States. Infect. Control Hosp. Epidemiol..

[6] Stock I., Grueger T., Wiedemann B. (2003). Natural antibiotic susceptibility of strains of *Serratia marcescens* and the *S. liquefaciens* complex: *S. liquefaciens* sensu stricto, *S. proteamaculans* and *S. grimesii*. Int. J. Antimicrob. Agents.

[7] Kowalski R.P., Kowalski T.A., Shanks R.M., Romanowski E.G., Karenchak L.M., Mah F.S. (2013). In vitro comparison of combination and monotherapy for the empiric and optimal coverage of bacterial keratitis based on incidence of infection. Cornea.

[8] Wall E., Majdalani N., Gottesman S. (2018). The Complex Rcs Regulatory Cascade. Annu. Rev. Microbiol..

[9] Steenhuis M., Ten Hagen-Jongman C.M., van Ulsen P., Luirink J. (2020). Stress-based high-througput screen assays to identify inhibitors of cell envelope biogenesis. Antibiotics.

[10] Erickson K.D., Detweiler C.S. (2006). The Rcs phosphorelay system is specific to enteric pathogens/commensals and activates *ydeI*, a gene important for persistent *Salmonella* infection of mice. Mol. Microbiol..

[11] Farris C., Sanowar S., Bader M.W., Pfuetzner R., Miller S.I. (2010). Antimicrobial peptides activate the Rcs regulon through the outer membrane lipoprotein RcsF. J. Bacteriol..

[12] Laubacher M.E., Ades S.E. (2008). The Rcs phosphorelay is a cell envelope stress response activated by peptidoglycan stress and contributes to intrinsic antibiotic resistance. J. Bacteriol..

[13] Konovalova A., Mitchell A.M., Silhavy T.J. (2016). A lipoprotein/b-barrel complex monitors lipopolysaccharide integrity transducing information across the outer membrane. Elife.

[14] Hirakawa H., Nishino K., Yamada J., Hirata T., Yamaguchi A. (2003). Beta-lactam resistance modulated by the overexpression of response regulators of two-component signal transduction systems in *Escherichia coli*. J. Antimicrob. Chemother..

[15] Lewis K. (2008). Multidrug tolerance of biofilms and persister cells. Curr. Top. Microbiol. Immunol..

[16] Stella N.A., Brothers K.M., Callaghan J.D., Passerini A.M., Sigindere C., Hill P.J., Liu X., Wozniak D.J., Shanks R.M.Q. (2018). An IgaA/UmoB-family protein from *Serratia marcescens* regulates motility, capsular polysaccharide, and secondary metabolite production. Appl. Environ. Microbiol..

[17] Clarke D.J. (2010). The Rcs phosphorelay: More than just a two-component pathway. Future Microbiol..

[18] Smith L.M., Jackson S.A., Malone L.M., Ussher J.E., Gardner P.P., Fineran P.F. (2021). The Rcs stress response inversely controls surface and CRISPR-Cas adaptive immunity to discriminate plasmids and phages. Nat. Microbiol..

[19] Brothers K.M., Callaghan J.D., Stella N.A., Bachinsky J.M., AlHigaylan M., Lehner K.L., Franks J.M., Lathrop K.L., Collins E., Schmitt D.M. (2019). Blowing epithelial cell bubbles with GumB: ShlA-family pore-forming toxins induce blebbing and rapid cellular death in corneal epithelial cells. PLoS Pathog..

[20] Di Venanzio G., Stepanenko T.M., Garcia Vescovi E. (2014). *Serratia marcescens* ShlA pore-forming toxin is responsible for early induction of autophagy in host cells and is transcriptionally regulated by RcsB. Infect. Immun..

[21] Pan X., Tang M., You J., Liu F., Sun C., Osire T., Fu W., Yi G., Yang T., Yang S.T. (2020). Regualtor RcsB controls prodigiosin synthesis and various cellular processes in *Serratia marcescens* JNB5-1. Appl. Environ. Microbiol..

[22] Romanowski E.G., Stella N.A., Romanowski J.E., Yates K.A., Dhaliwal D.K., St Leger A.J., Shanks R.M.Q. (2021). The Rcs stress response system regulator GumB modulates *Serratia marcescens* induced inflammation and bacterial proliferation in a rabbit keratitis model and cytotoxicity in vitro. Infect. Immun..

[23] Lin A., Rhee M.K., Akpek E.K., Amescua G., Farid M., Garcia-Ferrer F.J., Varu D.M., Musch D.C., Dunn S.P., Mah F.S. (2019). Bacterial Keratitis Preferred Practice Pattern(R). Ophthalmology.

[24] Wang D., Qi M., Calla B., Korban S.S., Clough S.J., Cock P.J., Sundin G.W., Toth I., Zhao Y. (2012). Genome-wide identification of genes regulated by the Rcs phosphorelay system in *Erwinia amylovora*. Mol. Plant-Microbe Interact..

[25] Bury-Moné S., Nomane Y., Reymond N., Barbet R., Jacquet E., Imbeaud S., Jacq A., Bouloc P. (2009). Global analysis of extracytoplasmic stress signaling in *Escherichia coli*. PLoS Genet..

[26] Boulanger A., Frances-Charlot A., Conter A., Castanié-Cornet M.-P., Cam K., Gutierrez C. (2005). Multistress regulation in *Escherichia coli*: Expression of *osmB* involves two independent promoters responding either to sigmaS or to the RcsCDB His-Asp phosphorelay. J. Bacteriol..

[27] Howery K.E., Clemmer K.M., Rather P.N. (2016). The Rcs regulon in *Proteus mirabilis*: Implications for motility, biofilm formation, and virulence. Curr. Genet..

[28] Huesa J., Giner-Lamia J., Pucciarelli M.G., Paredes-Martínez F., García del Portillo F., Marina A., Casino P. (2021). Structure-based analysis of *Salmonella* RcsB variants unravel new features of the Rcs regulon. Nucleic Acids Res..

[29] Hinchliffe S.J., Howard S.L., Huang Y.H., Clarke D.J., Wren B.W. (2008). The importance of the Rcs phosphorelay in the survival and pathogenesis of the enteropathogenic yersiniae. Microbiology.

[30] Lehner K.M., Stella N.A., Calvario R.C., Shanks R.M.Q. (2020). mCloverBlaster: A tool to make markerless deletions and fusion using lambda red and I-SceI in Gram-negative bacterial genomes. J. Microbiol. Methods.

[31] Cunha B.A. (2002). Antibiotic Essentials.

[32] Healy D.P., Holland E.J., Nordlund M.L., Dunn S., Chow C., Lindstrom R.L., Hardten D., Davis E. (2004). Concentrations of levofloxacin, ofloxacin, and ciprofloxacin in human corneal stromal tissue and aqueous humor after topical administration. Cornea.

[33] Tajima K., Miyake T., Koike N., Hattori T., Kumakura S., Yamaguchi T., Matsumoto T., Fujita K., Kuroda M., Ito N. (2014). In vivo challenging of polymyxins and levofloxacin eye drops against multidrug-resistant *Pseudomonas aeruginosa* keratits. J. Infect. Chemother..

[34] Protzko E., Bowman L., Abelson M., Shapiro A. (2007). Phase 3 safety comparisons for 1.0% azithromycin in polymeric mucoadhesive eye drops versus 0.3% tobramycin eye drops for bacterial conjuntivitis. Investig. Ophthalmol. Vis. Sci..

[35] Romanowski E.G., Romanowski J.E., Shanks R.M.Q., Yates K.A., Mammen A., Dhaliwal D.K., Jhanji V., Kowalski R.P. (2020). Topical vancomycin 5% is more efficacious than 2.5% and 1.25% for reducing viable methicillin-resistant *Staphylococcus aureus* in infectious keratitis. Cornea.

[36] Cahane M., Ben Simon G.J., Barequet I.S., Grinbaum A., Diamanstein-Weiss L., Goller O., Rubinstein E., Avni I. (2004). Human corneal stromal tissue concentration after consecutive doses of topically applied 3.3% vancomycin. Br. J. Ophthalmol..

[37] CLSI (2014). Performance Standards for Antimicrobial Susceptibility Testing 24th Informational Supplement.

[38] Lin Q.Y., Tsai Y.L., Liu M.C., Lin W.C., Hsueh P.R., Liaw S.J. (2014). *Serratia marcescens arn*, a PhoP-regulated locus necessary for polymyxin B resistance. Antimicrob. Agents Chemother..

[39] Romanowski E.G., Mah F.S., Yates K.A., Kowalski R.P., Gordon Y.J. (2005). The successful treatment of gatifloxacin-resistant *Staphylococcus aureus* keratitis with Zymar (gatifloxacin 0.3%) in a NZW rabbit model. Am. J. Ophthalmol..

[40] Kowalski R.P., Romanowski E.G., Mah F.S., Shanks R.M., Gordon Y.J. (2010). Topical levofloxacin 1.5% overcomes in vitro resistance in rabbit keratitis models. Acta Ophthalmol..

[41] Kowalski R.P., Romanowski E.G., Yates K.A., Romanowski J.E., Grewal A., Bilonick R.A. (2018). Is there a role for topical penicillin treatment of *Staphylococcus aureus* keratitis based on elevated corneal concentrations?. J. Clin. Ophthalmol. Optom..

[42] McDermott A.M. (2013). Antimicrobial compounds in tears. Exp. Eye Res..

[43] Pearlman E., Sun Y., Roy S., Karmakar M., Hise A.G., Szczotka-Flynn L., Ghannoum M., Chinnery H.R., McMenamin P.G., Rietsch A. (2013). Host defense at the ocular surface. Int. Rev. Immunol..

[44] Vasilchenko A.S., Rogozhin E.A. (2019). Sub-inhibitory effects of antimicrobial peptides. Front. Microbiol..

[45] Poole K. (2012). Bacterial stress responses as determinants of antimicrobial resistance. J. Antimicrob. Chemother..

[46] Flores-Kim J., Darwin A.J. (2014). Regulation of bacterial virulence gene expression by cell envelope stress responses. Virulence.

[47] Laloux G., Collet J.F. (2017). Major Tom to ground control: How lipoproteins communicate extracytoplasmic stress to the decision center of the cell. J. Bact..

[48] Macritchie D.M., Raivio T.L. (2009). Envelope Stress Responses. EcoSal Plus.

[49] Bruna R.E., Molino M.V., Lazzaro M., Mariscotti J.F., Garcia Véscovi E. (2018). CpxR-dependent thermoregulation of *Serratia marcescens* PrtA metalloprotease expression and its contribution to bacterial biofilm formation. J. Bacteriol..

[50] Qin H., Liu Y., Cao X., Jiang J., Lian W., Qiao D., Xu H., Cao Y. (2020). RpoS is a peiotropic regulator of motility, biofilm formation, exoenzymes, siderophore and prodigiosin production, and trade-off during prolonged stationary phase in *Serratia marcescens*. PLoS ONE.

[51] Sun Y., Wang L., Pan X., Osire T., Fang H., Zhang H., Yang S.T., Yang T., Rao Z. (2020). Improved prodigiosin production by relieving CpxR temperature-sensitive inhibition. Front. Bioeng. Biotechnol..

[52] Wilf N.M., Salmond G.P. (2012). The stationary phase sigma factor, RpoS, regulates the production of a carbapenem antibiotic, a bioactive prodigiosin and virulence in the enterobacterial pathogen *Serratia* sp. ATCC 39006. Microbiology.

[53] Steenhuis M., Corona F., Ten Hagen-Jongman C.M., Vollmer W., Lambin D., Selhorst P., Klaassen H., Versele M., Chatltin P., Luirink J. (2021). Combining cell envelope stress reporter assays in a screening approach to identify BAM complex inhibitors. ACS Infect. Dis..

[54] Bertani G. (1951). Studies on lysogenesis. I. The mode of phage liberation by lysogenic *Escherichia coli*. J. Bacteriol..

[55] Shanks R.M., Stella N.A., Kalivoda E.J., Doe M.R., O’Dee D.M., Lathrop K.L., Guo F.L., Nau G.J. (2007). A *Serratia marcescens* OxyR homolog mediates surface attachment and biofilm formation. J. Bacteriol..

[56] Romanowski E.G., Lehner K.M., Martin N.C., Patel K.R., Callaghan J.D., Stella N.A., Shanks R.M.Q. (2019). Thermoregulation of prodigiosin biosynthesis by *Serratia marcescens* is controlled at the transcriptional level and requires HexS. Pol. J. Microbiol..

[57] Shanks R.M., Caiazza N.C., Hinsa S.M., Toutain C.M., O’Toole G.A. (2006). *Saccharomyces cerevisiae*-based molecular tool kit for manipulation of genes from gram-negative bacteria. Appl. Environ. Microbiol..

[58] Shanks R.M., Kadouri D.E., MacEachran D.P., O’Toole G.A. (2009). New yeast recombineering tools for bacteria. Plasmid.

[59] Callaghan J.D., Stella N.A., Lehner K.M., Treat B.R., Brothers K.M., St Leger A.J., Shanks R.M.Q. (2020). Generation of Xylose-inducible promoter tools for *Pseudomonas* species and their use in implicating a role for the Type II secretion system protein XcpQ in inhibition of corneal epithelial wound closure. Appl. Environ. Microbiol..

