# Supplementary material for: Antibiotics Used in Empiric Treatment of Ocular Infections Trigger the Bacterial Rcs Stress Response System Independent of Antibiotic Susceptibility
Source: Antibiotics (Basel). 2021 Aug 25;10(9):1033. doi: 10.3390/antibiotics10091033 (PMC8470065; doi:10.3390/antibiotics10091033)
Supplement: Supplementary file 1 [file antibiotics-10-01033-s001.zip › antibiotics-1318979-supplementary.pdf]

Supplemenary Figure for Harshaw, et al. 2021

From Antibiotics used in empiric treatment of ocular infections trigger the bacterial Rcs stress response system independent of antibiotic susceptibility

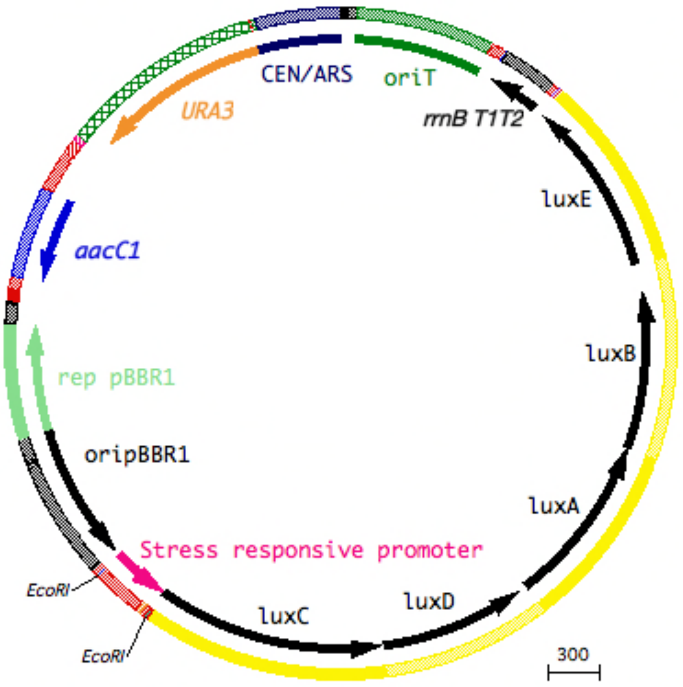

Figure S1. Diagram of pMQ747 used in this study. Starting from the origin, the origin of conjugal transfer, oriT, is from RP4; the *rrnB* T1T2 are transcriptional terminators from *Escherichia coli*. The *luxCDABE* operon provides luminescence. The stress responsive promoter was altered in each of the plasmids used in this study. The oripBBR1-rep pBBR1 is a broad host-range medium copy replicon that is sufficient for replication in a large number of Gram-negative bacterial species. The *aacC-1* gene provides gentamicin resistance, and the *URA3* and *CEN/ARS* portions allow replication in *Saccharomyces cerevisiae* for recombineering purposes. The size bar represents 300 base pairs.

Supplemental tables for Antibiotics used in empiric treatment of ocular infections trigger the bacterial Rcs stress response system independent of antibiotic susceptibility

Nathaniel S. Harshaw, Nicholas A. Stella, Kara M. Lehner, Eric G. Romanowski, Regis P. Kowalski, and Robert M.Q. Shanks

Table S1. *S. marcescens* strains and plasmids used in this study.

Strain	Description	Source
K904	Wild-type *S. marcescens* keratitis isolate	[1]
Δ*gumB*	K904 with deletion of *gumB* gene	[2]
Δ*rcsB*	K904 with *rcsB* ORF replaced by *mClover*	[3]
Δ*gumB* Δ*rcsB*	K904 with deletion mutations of *gumB* and *rcsB*	[3]
S17-1 λ-*pir*	*Escherichia coli* conjugal plasmid donor strain	[4]
pMQ414	pBBR1-replicon plasmid with *nptII* promoter driving *tdtomato* expression	[5]
pMQ589	RSF1010-IncQ plasmid with *nptII* promoter driving *luxCDABE* expression	This study
pMQ670	pBBR1-replicon plasmid with *xut* promoter driving *luxCDABE* expression	[6]
pMQ713	pBBR1-replicon plasmid with *pigA* promoter - *luxCDABE* expression	[7]
pMQ747	pMQ713 with SMDB11_1637 promoter -*luxCDABE*	This study
pMQ748	pMQ713 with SMDB11_2817 promoter -*luxCDABE*	This study
pMQ749	pMQ713 with SMDB11_1194 promoter –*luxCDABE*	This study

Table S2. Oligonucleotides used in this study.

Name of sequence	Sequence (5' to 3')
3805	agaccgcttctgcgttctgatttaatctgtatcaGGATCCTCAACTATCAAACGCTTCGG
3806	gatcaagatctgatcaagagacaggatgaggaGAATTCATGACTAAAAAAATTTCATTCA
4858<br>SMDB11_1637	ttgcactaaatcatcactttcgggaaagatttcaacctggccgttaatgatgaatgaaatctttttagtcatGAATTCTTCCTGGTAAATTGGCTGATTAATGGATTAAAAATAATCCGATTAACCCACCGGTAGAAGTTGGGATGAATGTATTCCTCCGAAAGAAAATCGAACAGGTAAAAATTCTTAATTTTTCGGCGGTGAAAAGCGATTGCTCGCCGAATGTGCGGCCACCGACAAAACCGTGGGTCTATACTGGGAAGACGCCTCGCGGGGTGCGGGGCACACACTCTGGAGGCCGCCATGTCCGATCTCGTTTCCGCGTCCGGCAAACCGGTGAAAATCCCCGGCCCGGACCACCCAATCACCTTGACCCGCCATCCGGCGCGGGTTGTCGTGCGTGCTGCCGGTCAGACCCTGGCCGACAGTCGCAGAATTCgtcgactccagtcgggaaacctgtcgtgccagctgcattaatgcgccctacgggcttgct
4860<br>SMDB11_2817	ttgcactaaatcatcactttcgggaaagatttcaacctggccgttaatgatgaatgaaatctttttagtcatGAATTCCTTCTCCTTCCCGCTTGCGCGGCCTGGCCGTTTGTCACTTTATGAAGCGCGTTCATCAGTTTAAAAAAGCGGCCTTATCGTTATTTTGCTGCAATATCGGCATCATAGCGCCGGCGCATAGGGTTGAATCTGAGAGCCACTCTGAAAATGATGCAGGAGGTTAAGATTTGAGATTTTTCTGGAGTATCGCCGCTCGATTGAACGGCGGAATTTGTAAGCGATTACAATAAGATGTTCTCGATCTGCGCGAGCTCTTCTTCACTGAAGTGACGATTGGCCAGCATGCCCACCGCGTCTTCTATCTGCGCATTCTTGCTGGCGCCGATCAGCACTGAGGGAATTCgtcgactccagtcgggaaacctgtcgtgccagctgcattaatgcgccctacgggcttgctctccgggcttcgccctg
4862<br>SMDB11_1194	gattgcactaaatcatcactttcgggaaagatttcaacctggccgttaatgatgaatgaaatctttttagtcatGAATTCAGGAAACCTCCTGAAAGTGTTACCACGCATTTTTTCCGGGGTCTGTTACCGGCATGGCACCGCGCTTATTATTTTGCGCATGGGCGGCTTGCGGTATCCCGGTTTACGGCGTGCGCGACGCGCCGTCTGTAAACGAGGTAAAGTCTGCACAATGATTTGAGGGCTCGCAATAAGACAAGGGGACCAAAAATGCGGGAAAACGGCCGTTTTGCCGTTGTTTAGGAGGAATCTTAAGAATTTTACCCATGTCGGCGACAGCTTATGCTGAAGAATCAAGCAACCGCCGCCGTTAGCCTCGCCCCGCCATAAAAAAACCCGCGCAGGCGAACCGGCGCGGGGAATTCgtcgactccagtcgggaaacctgtcgtgccagctgcattaatgcgccctacgggcttgctctccgggcttcgccc

Lower case sequence represents DNA for targeting recombination, upper case is for priming or promoter sequence. Underlined sequence indicates an introduced restriction enzyme cleavage site.

References

1. Kalivoda, E.J.; Stella, N.A.; Aston, M.A.; Fender, J.E.; Thompson, P.P.; Kowalski, R.P.; Shanks, R.M. Cyclic AMP negatively regulates prodigiosin production by *Serratia marcescens*. *Res Microbiol* **2010**, *161*, 158-167, doi:S0923-2508(09)00256-3 [pii]10.1016/j.resmic.2009.12.004.
2. Stella, N.A.; Brothers, K.M.; Callaghan, J.D.; Passerini, A.M.; Sigindere, C.; Hill, P.J.; Liu, X.; Wozniak, D.J.; Shanks, R.M.Q. An IgaA/UmoB-family protein from *Serratia marcescens* regulates motility, capsular polysaccharide, and secondary metabolite production. *Appl Environ Microbiol* **2018**, *84*, pii: e02575-02517, doi:10.1128/AEM.02575-17.
3. Lehner, K.M.; Stella, N.A.; Calvario, R.C.; Shanks, R.M.Q. mCloverBlaster: a tool to make markerless deletions and fusion using lambda red and I-SceI in Gram-negative bacterial genomes. *J Microbiol Methods* **2020**, *178*, doi:10.1016/j.mimet.2020.106058.
4. Miller, V.L.; Mekalanos, J.J. A novel suicide vector and its use in construction of insertion mutations: osmoregulation of outer membrane proteins and virulence determinants in *Vibrio cholerae* requires *toxR*. *J Bacteriol* **1988**, *170*, 2575, doi:DOI: 10.1128/jb.170.6.2575-2583.1988.
5. Mukherjee, S.; Brothers, K.M.; Shanks, R.M.; Kadouri, D.E. Visualizing *Bdellovibrio bacteriovorus* by Using the tdTomato Fluorescent Protein. *Appl Environ Microbiol* **2015**, *82*, 1653-1661, doi:10.1128/AEM.03611-15.
6. Callaghan, J.D.; Stella, N.A.; Lehner, K.M.; Treat, B.R.; Brothers, K.M.; St Leger, A.J.; Shanks, R.M.Q. Generation of Xylose-inducible promoter tools for *Pseudomonas* species and their use in implicating a role for the Type II secretion system protein XcpQ in inhibition of corneal epithelial wound closure. *Appl Environ Microbiol* **2020**, doi:10.1128/AEM.00250-20.
7. Romanowski, E.G.; Lehner, K.M.; Martin, N.C.; Patel, K.R.; Callaghan, J.D.; Stella, N.A.; Shanks, R.M.Q. Thermoregulation of prodigiosin biosynthesis by *Serratia marcescens* is controlled at the transcriptional level and requires HexS. *Pol J Microbiol* **2019**, *68*, 43-50, doi:doi: 10.21307/pjm-2019-005.
